# Association of glutathione S-transferase T1 and M1 polymorphisms with prostate cancer susceptibility in populations of Asian descent: a meta-analysis

**DOI:** 10.18632/oncotarget.5346

**Published:** 2015-09-24

**Authors:** Da-Long Cao, Ding-Wei Ye, Bo Dai, Hai-Liang Zhang, Yi-Jun Shen, Yao Zhu, Yi-Ping Zhu, Guo-Hai Shi, Chun-Guang Ma, Wen-Jun Xiao, Xiao-Jian Qin, Guo-Wen Lin

**Affiliations:** ^1^ Department of Urology, Fudan University Shanghai Cancer Center, Shanghai, 200032, China; ^2^ Department of Oncology, Shanghai Medical College, Fudan University, Shanghai, 200032, China

**Keywords:** prostate cancer, susceptibility, polymorphism, glutathione S-transferases T1, glutathione S-transferases M1

## Abstract

**Background:**

Genetic polymorphism was hypothesized to be reason of variation in prostate cancer incidence among different racial group. Based on that published data on the association of prostate cancer susceptibility with polymorphisms in genes encoding Glutathione S-transferases (GSTs) were inconclusive, the aim of this study was to more precisely address the role of GSTs polymorphisms (especially, GSTT1 and GSTM1 deletions) on prostate cancer risk in Asian descent.

**Methods:**

A meta-analysis including 8 articles with 711 cases and 1122 controls for GSTT1 and 1098 cases and 1588 controls for GSTM1 was performed.

**Results:**

Significantly increased prostate cancer risk was found among subjects carrying GSTM1 null genotype (odds ratio (OR) = 1.403; 95% confidence interval (CI) = 1.088 – 1.808) but not among subjects carrying GSTT1 deletion genotype (OR = 0.959; 95%CI = 0.709 – 1.297). When stratified by country, the null genotype of GSTT1 neither increased nor decreased prostate cancer risk significantly in China (OR = 1.355; 95%CI = 0.895 – 2.049), Japan (OR = 0.812; 95%CI = 0.545 – 1.211), and Korea (OR = 1.056; 95%CI = 0.727 – 1.534). While significant association of elevated prostate cancer risk with GSTM1 deletion were found in China (OR = 1.665; 95%CI = 1.324 – .094) and Korea (OR = 1.914; 95%CI = 1.311 – 2.793) but not in Japan (OR = 0.980; 95%CI = 0.726 – 1.321).

**Conclusion:**

In summary, this meta-analysis suggested that the null genotype of GSTM1 rather than GSTT1 may be involved in the etiology of prostate cancer in Asian population.

## INTRODUCTION

Prostate cancer is the most commonly diagnosed malignancy and the second leading cause of cancer-related deaths in the western male population [1]. In Asian, especially in china with the largest population in world, the detection rate and thus incidence of prostate cancer is increasing rapidly due to the extension of life expectancy, the change of lifestyles and the improvement of clinical skills [2]. Prostate cancer has become a major global public health problem, but it affects individuals of Asian ancestry less than their white and African counterparts [3, 4]. Additionally, individuals of Asian descent who live in diverse environment around the world still have low risk of developing prostate cancer. Therefore, some genetic factors represented individual characteristics may contribute to the mechanism for prostate carcinogenesis among different race.

Low-penetrance susceptibility genes combining with environmental factors may be crucial in the development of cancer [5]. Polymorphisms in genes (e.g. susceptibility genes) responsible for the metabolism of environmental and endogenous carcinogens could be associated with prostate cancer and thus these polymorphisms could be used as biomarkers for detecting men at risk of prostate cancer [6]. To date, some genes possessing detoxification and polymorphism have been identified as potential susceptibility genes for prostate cancer. One of the most promising candidates is the Glutathione S-transferases (GST) family, which catalyze reactions between the cytosolic glutathione and electrophilic compounds to eliminate local accumulation of electrophilic carcinogens and thus protect against carcinogenesis [7, 8]. Among the GSTs isoforms, the polymorphism of Glutathione S-transferase T1 and M1 (GSTT1 and GSTM1) to prostate cancer risk has become a research focus in scientific community and has drawn increasing attention.

The roles of GSTT1 and GSTM1 polymorphism in prostate cancer risk have been studied previously, but most published results are limited to western populations. Up to now, several original studies have reported the relationship between these polymorphism and prostate cancer risk in men of Asian descent [9–16]; however, the results are inconclusive, partially due to the possible limited effect of the polymorphism on prostate cancer risk and the relatively small sample size in each of published studies. Therefore, we perform this meta-analysis here to obtain a more precise estimation of these associations in individuals of Asian descent.

## RESULTS

### Study characteristics

**Figure 1:**
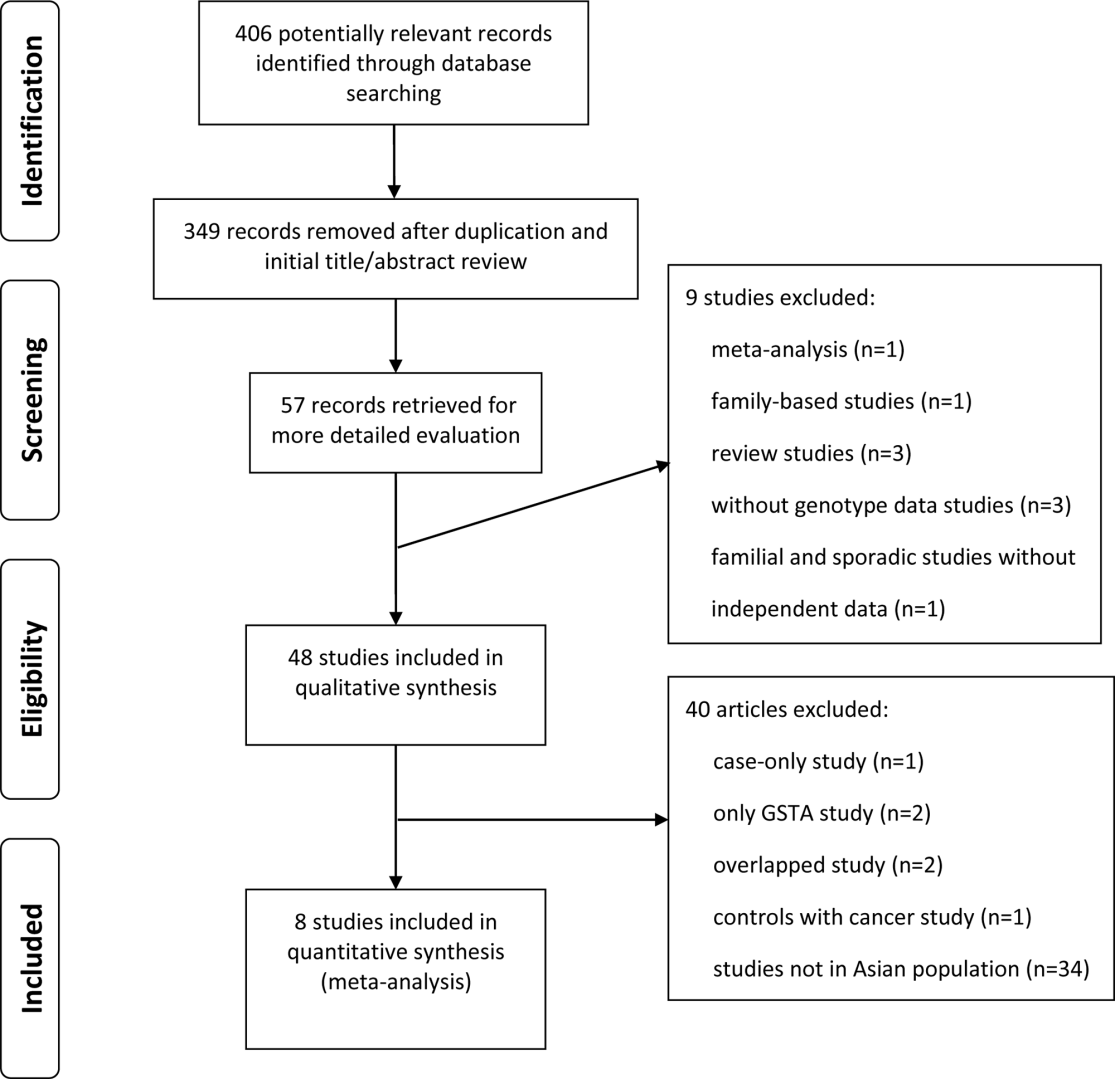
Literature Flowchart

As showed in Figure 1, a total of 8 studies including 711 cases and 1122 controls for GSTT1 and 1098 cases and 1588 controls for GSTM1 were coincided with the predetermined inclusion criteria. All of these included studies were hospital-based, of which 4 from China, 3 from Japan, 1 from Korea. Table 1 lists the studies involved and their main characteristics.

**Table 1:** Main characteristics of eligible studies included in the meta-analysis

References	Publication of year	Country	Ethnicity	Design	GSTT1		GSTM1	
					Case (n/p)	Control (n/p)	Case (n/p)	Control (n/p)
Kwon et al	2011	Korea	Asian	HB	85/81	163/164	90/76	125/202
Li et al	2008	China	Asian	HB	-	-	121/87	96/134
Yang et al	2006	China	Asian	HB	89/74	95/107	99/64	112/90
Komiya et al	2005	Japan	Asian	HB	74/112	139/149	93/93	157/131
Guan et al	2005	China	Asian	HB	-	-	48/35	48/67
Lai et al	2005	Taiwan	Asian	HB	-	-	57/39	55/66
Nakazato et al	2003	Japan	Asian	HB	40/41	44/61	38/43	53/52
Murata et al	2001	Japan	Asian	HB	47/68	104/96	57/58	85/115

Abbreviations: HB, hospital-based study; GSTT1, Glutathione S-transferase T1; GSTM1, Glutathione S-transferase M1; n/p, null/present.

### GSTT1 deletion and prostate cancer

After all the extracted data were pooled, a total of 711 cases and 1122 controls were available for GSTT1 analysis. As detailed in Table 2, there didn't exist significant relationship between the null genotype of GSTT1 and increased prostate cancer risk (OR = 0.959; 95%CI = 0.709 – 1.297). Statistically significant heterogeneity across studies was observed in the analysis (I^2^ = 58.9%; *p* value = 0.045). It's clear that this analysis is based on pooling data from different country. When subgroup analyses were conducted in different country's group, the null genotype of GSTT1 also neither increased nor decreased the risk of prostate cancer significantly in China, Japan, and Korea (OR = 1.355, 95%CI = 0.895 – 2.049; OR = 0.812, 95%CI = 0.545 – 1.211; OR = 1.056, 95%CI = 0.727 – 1.534; respectively).

**Table 2:** Summary of meta-analysis for the association of GSTT1, GSTM1 deletion with prostate cancer risk in Asian population

	N	Case	Control	OR (95% CI)	*P*_h_ value (I^2^)
**GSTT1 (null vs. present)**					
China including Taiwan	1	163	202	1.355 (0.895–2.049)	-
Japan	3	382	593	0.812(0.545 – 1.211)	0.111 (54.5%)
Korea	1	166	327	1.056 (0.727 – 1.534)	-
Total	5	711	1122	0.959 (0.709 – 1.297)	0.045 (58.9%)
**GSTM1 (null vs. present)**					
China including Taiwan	4	550	668	1.665(1.324 – 2.094)	0.431 (0.0%)
Japan	3	382	593	0.980(0.726 – 1.321)	0.275 (22.5%)
Korea	1	166	327	1.914(1.311 – 2.793)	-
Total	8	1098	1588	1.403(1.088 – 1.808)	0.013 (60.7%)

Abbreviations: GSTT1, Glutathione S-transferase T1; GSTM1, Glutathione S-transferase M1; N, number of study; OR, odds ratio; 95%CI, 95% confidence interval; *P*_h_ value, *P* value of Q-test for heterogeneity test; I^2^, I squared in test of heterogeneity.

Random-effects model was used when *P* value < 0.1 for heterogeneity test; otherwise, fixed-effects model was used.

### GSTM1 deletion and prostate cancer

The data of 1098 cases and 1588 controls derived from pooled data were usable for meta-analysis of GSTM1. In Table 2, the results suggested that GSTM1 null genotype did increase the risk of prostate cancer in Asian population (OR = 1.403; 95%CI = 1.088 – 1.808), with statistically significant heterogeneity among studies (I^2^ = 60.7%; *p* value = 0.013). Then, analyses stratified by different country showed a significant association of elevated prostate cancer risk with GSTM1 deletion in China and Korea, the ORs were 1.665 (95%CI = 1.324 – 2.094) and 1.914 (95%CI = 1.311 – 2.793) respectively. However, such significant connection between GSTM1 null genotype and increased prostate cancer risk were not present in Japan subjects (OR = 0.980; 95%CI = 0.726 – 1.321).

### Sensitivity analysis

**Figure 2:**
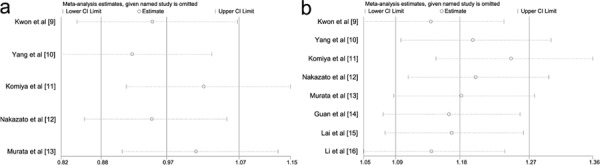
Sensitivity analysis of this meta-analysis The vertical axis indicates the log odds ratio of GSTT1 deletion variant **2a.** and GSTM1 deletion variant **2b.** that have been estimated. GSTT1, Glutathione S-transferase T1; GSTM1, Glutathione S-transferase M1.

For GSTT1 deletion genotype related to prostate cancer susceptibility, several studies [11, 13] might result in heterogeneity according to the results from sensitivity analysis (Figure 2a). When these studies of Komiya et al [11] and Murata et al [13] were omitted, non-significant relationship between GSTT1 polymorphism and prostate cancer was not materially affected (OR = 1.211, 95%CI = 0.943 – 1.555; I^2^ = 0.0%, *p* value for heterogeneity = 0.626). For GSTM1 null genotype, when deleting the study of Komiya et al [11] based on sensitivity analysis (Figure 2b), significant heterogeneity across studies was not detected (I^2^ = 30.1%; *p* value = 0.198). Importantly, statistically significant association of increased prostate cancer risk with GSTM1 deletion was still not substantially altered (OR = 1.567; 95%CI = 1.320 – 1.860). These evidences indicated that the present results were statistically robust.

### Publication bias

**Figure 3:**
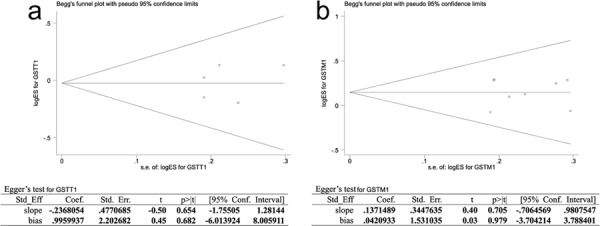
Publication bias of literatures for GSTT1 3a. and GSTM1 3b. were tested by Begg's funnel plot and Egger's test. GSTT1, Glutathione S-transferase T1; GSTM1, Glutathione S-transferase M1

Publication bias of literatures was evaluated using Begg's funnel plot and Egger's test. The shapes of the funnel plots for GSTT1 and GSTM1 seemed to be symmetrical, but the extent of symmetry couldn't be obtained quantificationally. Therefore, Egger's test was applied to provide statistical evidence of funnel plot symmetry, and the results still did not reveal any evidence of publication bias (Figure 3a–3b).

## DISCUSSION

The present meta-analysis from 8 studies mainly evaluated the potential role of GSTT1 and GSTM1 polymorphisms in prostate cancer susceptibility in Asian population.

Variation in prostate cancer incidence among different racial groups and geographic locations has been well documented [3, 4]. For etiology, both environmental and genetic factors influence the carcinogenesis of prostate cancer [22]. Meanwhile, it is well recognized that individual susceptibility to the same kind of cancer is still exist even with the same environmental exposure. Therefore, this diversity may be mainly explained by host factors, especially genetic polymorphism involved in carcinogenesis. These information show clearly that a strategy of investigating genetic polymorphism would be essential in exploring prostate cancer susceptibility. In the field of life science, increasing numbers of studies have reported that the polymorphisms of GST family, especially GSTT1 and GSTM1, were emerging as important susceptibility in prostate carcinogenesis. Based on the evidences mentioned above, it was necessary to perform a systematic analysis for better understanding of the relationship between these polymorphisms and prostate cancer risk in Asian population.

The current study through meta-analysis indicated that significantly elevated prostate cancer risk was associated with GSTM1 rather than GSTT1 null genotype. As statistically significant between-study heterogeneity was found when carrying out meta-analysis for both GSTT1 and GSTM1, subgroups analyses in the different country were conducted. Then we found similar non-significant relationship between GSTT1 deletion and prostate cancer risk among in china, Korea, and Japan, while significant association of null genotype of GSTM1 with increased prostate cancer risk was detected in China and Korea but not in Japan. When studies led to heterogeneity were removed according to sensitivity analysis, the pooled ORs of both GSTT1 and GSTM1 were not substantially altered, indicating that the present results were statistically robust. These results were similar to some previous studies and simultaneously inconsistent to other studies [23, 24]. The inconsistent results among these studies might be explained by ethnic differences [25].

In the GST family, GSTM1 has been proved to directly implicate in DNA adduct formation caused by benzo(a)pyrene, the main component of cigarette smoke [26]; GSTT1 is involved in conjugating smaller molecules (e.g. epoxides) and thus involved in oxidative processes such as those caused by inflammation [27]. The null genotype of GSTM1 eliminates the gene function, leading to the inability to eliminate electrophilic carcinogens as efficiently and thus increase prostate cancer risk. However, non-significant relationship between GSTT1 deletion genotype and prostate cancer carcinogenesis were found in our meta-analysis. This result implied a role of functional GSTT1 in prostate carcinogenesis based on that GSTT1 might also be involved in activating compounds acted as prostate carcinogens [28], which could derive from exposures to endogenous hormones or exogenous toxins that are specific to certain geographic areas. In addition, the effect of GSTT1 deletion might be blinded by the presence of other unidentified causal factors involved in prostate carcinogenesis in Asian. Furthermore, the obtained results for GSTT1 and GSTM1 in our meta-analysis might be unacceptable due to chance because studies with small sample size might result in insufficient statistical power to detect a slight effect or might generate a fluctuated risk estimate [29]. Considering the limited studies and the number of Asian population involved in the meta-analysis, our results should be interpreted with caution.

The limitations of this meta-analysis should be acknowledged. Firstly, only published studies were included while studies with null or unexpected results had a lower probability of being published and weren't included in meta-analysis, thus bias may have appeared. Secondly, all of studies included in our systematic analysis were the hospital-based studies which inherently have selection biases because such controls may not represent the general population, particularly when the genotypes under investigation were associated with the disease-related conditions that the hospital-based controls may posses. Thirdly, heterogeneity among studies was existed, which may be derive from the study design, the source of controls, the differences of genetic background, and the environment presented among different country. Fourthly, the shortage of funnel plots for evaluating publication bias was not avoided, as the reported reasons that the probability of exactly detecting publication bias through funnel plot was equivalent to a coin toss [30] and the funnel plot was misleading [31]. Fifthly, the overall results were based on unadjusted estimates, while a more precise evaluation need to be adjusted by other co-variants if available including age, PSA (prostate specific antigen) level, smoking status, drinking status, obesity, environmental factors, and other lifestyle. Finally, meta-analysis remains as retrospective research which is subjected to the methodological deficiencies of the involved studies.

Despite some limitations, this meta-analysis suggested that the GSTM1 null genotype was associated with enhanced risk of prostate cancer carcinogenesis, but non-significant association was observed for GSTT1 deletion with prostate cancer risk. However, high-quality epidemiological studies using large sample, standardized unbiased genotyping methods, homogeneous prostate cancer patients and well matched controls and considering gene-gene and gene-environment interactions would be necessary to conduct in the future. Such studies taking these factors mentioned above into account might eventually provide our better, comprehensive understanding of the association of the polymorphism of GST family (including GSTT1 and GSTM1) with prostate cancer susceptibility in Asian even worldwide population.

## MATERIALS AND METHODS

### Publication search

A comprehensive systematic search through Medline, PubMed, Embase, and Web of Science was conducted for all medical publications up to May, 2014. The search strategy was performed using the search terms “prostate”, “polymorphism”, “Glutathione S-transferase”, “GST”, “Glutathione S-transferase T1”, “GSTT1”, “Glutathione S-transferase M1” and “GSTM1” without language restriction. After all searched studies were retrieved, the reference lists of their papers and review articles were checked and hand-searched to ascertain additional undetected but eligible studies. Only published studies with full-text papers, rather than any meeting or conference abstract, were included. When more than one of the same patient population was included in several articles, only the most recent or complete study was used in this meta-analysis.

### Inclusion criteria and exclusion criteria

The following inclusion criteria were considered: (a) case-control or cohort studies using Asian population that had original data for a quantitative evaluation of the association of GSTT1 and/or GSTM1 present/null polymorphism with prostate cancer; (b) an appropriate description of GSTT1, and GSTM1 polymorphisms in cases and controls; (c) cases with prostate cancer were eligible regardless of whether they had a first-degree relative with prostate cancer or not, regardless of tumor stage; (d) controls were eligible if they were male, with or without BPH, or other diseases; (e) sufficient published data for calculating an odds ratio (OR) with 95% confidence interval (CI).

The exclusion criteria were: (a) studies without the raw data of genotype of GSTM1, and GSTT1; (b) case-only studies, family-based studies, case reports, editorials, and review articles (including meta-analyses); (c) studies that compare the racial variation of GSTT1 and/or GSTM1 variants in healthy population; (d) controls with any type of tumor; (e) studies that used GSTT1 and/or GSTM1 polymorphisms to predict survival in prostate cancer; (f) studies that investigated GSTT1 and/or GSTM1 variants as makers for response to therapy.

### Data extraction

According to the inclusion/exclusion criteria mentioned above, information was carefully extracted from all eligible articles independently by two of the authors. Any disagreement was resolved by discussion between the two authors. If these two authors could not reach a consensus, another author was consulted to resolve the dispute and a final decision was made by the majority of the votes. For each study, the following data were reviewed and collected: first author’ name, year of publication, the country of origin, ethnicity of the study population, subject source, involved genes, total number of cases and controls, and numbers of cases and controls with the present/null genotypes. Subject source were classified to population-based studies and hospital-based studies. We did not define any minimum number of patients to include in this meta-analysis.

### Statistical Analyses

The strength of relationship between the GSTT1 and/or GSTM1 present/null polymorphism and prostate cancer susceptibility was evaluated using crude ORs with 95%CI. The pooled ORs were performed for null versus present genotype. Heterogeneity assumption was tested by the chi-square-based Q-test [17]. A *p* value > 0.10 for the Q-test indicated a lack of heterogeneity among studies, so the pooled OR estimate of each study was calculated by the fixed-effects model (the Mantel-Haenszel method)[18]. Otherwise, the random-effects model (the DerSimonian and Laird method) was used [19]. The stability of the results was assessed using sensitivity analysis, which repeats the meta-analysis while sequentially omitting single study each time to evaluate the influence of each study on the pooled OR [20]. An estimate of potential publication bias was carried out by Begg's funnel plot, in which the standard error of log (OR) of each study was plotted against its log (OR). Funnel plot asymmetry, which suggested a possible publication bias, was assessed by the method of Egger's linear regression test, a linear regression approach to measure funnel plot asymmetry on the natural logarithm scale of the OR. A *p* value less than 0.05 from *t*-test suggested by Egger was considered representative of statistically significant publication bias [21]. All the statistical tests were performed using the software of STATA version 11.0 (Stata Corporation, College Station, TX).
